# Pilocytic Astrocytoma Causing Brainstem Compression in Pregnancy: Case Report with Literature Review

**DOI:** 10.3390/neurolint18030043

**Published:** 2026-02-25

**Authors:** Muratbek A. Tleubergenov, Daniyar K. Zhamoldin, Nurzhan A. Ryskeldiyev, Aigul D. Tolepbergenova, Aisa Z. Nurpeisov, Zhanat T. Takenov, S. Akshulakov

**Affiliations:** 1 Tsoi G.B. Scientific and Educational Center of Surgery, NCJSC “Astana Medical University”, Astana 010000, Kazakhstan; 2 Department of Brain Neurosurgery, JSC “National Centre for Neurosurgery”, Astana 010000, Kazakhstan; nurjan.ryskeldiev@ncn.kz (N.A.R.); aigul.tolepbergenova@ncn.kz (A.D.T.); aisa.nurpeisov@ncn.kz (A.Z.N.); janat.takenov@ncn.kz (Z.T.T.); serik.akshulakov@ncn.kz (S.A.)

**Keywords:** pilocytic astrocytoma, pregnancy, brainstem compression, posterior fossa tumour, multidisciplinary management, fetal monitoring

## Abstract

**Background:** Primary central nervous system tumours in pregnancy are exceptionally rare, with posterior fossa lesions presenting particular diagnostic and management challenges due to their confined anatomical location and proximity to critical neurovascular structures. Pilocytic astrocytoma (PA), typically a paediatric tumour, is uncommon in adults and exceedingly rare in pregnant patients. The physiological changes in pregnancy can obscure tumour-related symptoms, contributing to diagnostic delay and increased maternal–fetal risk. **Methods:** We report the case of a 24-year-old pregnant woman at 23 weeks and 5 days’ gestation who presented with progressive neurological deterioration secondary to a cystic mass in the right cerebellar hemisphere. MRI revealed significant brainstem compression and triventricular hydrocephalus. **Results:** A multidisciplinary team performed an urgent retrosigmoid craniotomy with gross total tumour resection under general anaesthesia and continuous intraoperative fetal monitoring. Histopathology confirmed PA (CNS WHO Grade I). Postoperative recovery was uneventful, and both maternal and fetal outcomes were favourable. **Conclusions:** This case highlights the importance of early neuroimaging, multidisciplinary coordination, and timely surgical intervention in managing posterior fossa tumours during pregnancy. Although PAs are considered low-grade gliomas, their behaviour in pregnancy can be unpredictable. With careful perioperative planning, neurosurgical treatment can be safely undertaken during gestation, offering optimal outcomes for both mother and fetus.

## 1. Introduction

Primary tumours of the CNS arising during pregnancy are exceptionally rare, with an estimated incidence of approximately 15 cases per 100,000 live births [1]. Their clinical presentation is frequently obscured by the physiological changes in gestation—such as nausea, vomiting, headache, and dizziness—thereby contributing to diagnostic delays and increasing the risk of neurological decline. Management in such complex scenarios necessitates a highly individualised, multidisciplinary approach involving neurosurgery, obstetrics, neurology, and anaesthesiology [2,3].

Tumours localised to the posterior cranial fossa are particularly uncommon and present significant maternal and fetal hazards due to their predilection for causing obstructive hydrocephalus and compression of the brainstem structures [4]. Expansive lesions such as astrocytomas and medulloblastomas often require prompt surgical intervention to alleviate intracranial pressure and preserve neurological function. Notwithstanding the inherent perioperative risks, recent advancements in intraoperative neuronavigation and enhanced modalities for fetal monitoring have contributed to the growing feasibility of neurosurgical procedures during pregnancy [5].

The relationship between glioma progression and gestation remains incompletely understood. Several reports have indicated that the growth kinetics—specifically the velocity of diametric expansion—of low-grade gliomas (LGGs) may be accelerated during pregnancy, potentially mediated by hormonal, vascular, and growth-related changes associated with the gravid state [6,7].

The present case is of particular interest due to its rarity: a cerebellar PA exhibiting a slow progression but resulting in significant brainstem compression during the second trimester, manifesting with severe neurological compromise that required urgent surgical decompression. The favourable maternal and fetal outcomes achieved in this context underscore the clinical relevance of such cases, which remain sparsely documented in the existing literature.

## 2. Case Description

### 2.1. Demographics of the Patient

A 24-year-old pregnant woman (G1P0) at 23 weeks and 5 days of gestation presented with a 5–6-month history of progressive neurological symptoms, including headache, dizziness, nausea, vomiting, photophobia, general malaise, and difficulty walking independently. She also reported decreased vision in the right eye and a sensation of facial numbness. These symptoms were initially attributed to pregnancy and managed conservatively during multiple inpatient admissions without significant improvement.

Over the 2–3 weeks prior to presentation, her condition worsened, with an increased frequency of vomiting, further visual impairment in the right eye (described as “clouding”), and worsening facial numbness. Given the progression despite conservative therapy, a neurologist recommended a brain MRI. Following imaging, the patient was urgently referred to the emergency department of the regional clinical hospital for further evaluation and management. From there, she was transported by Sanaviation to the National Centre for Neurosurgery in Astana, Kazakhstan, for further evaluation and management.

### 2.2. Investigation

Upon admission, the patient was alert, with a Glasgow Coma Scale score of 14. Verbal communication was limited, and mild impairments in insight and awareness of her clinical condition were noted. She was oriented to person, place, and time but exhibited psychomotor slowing and rapid mental fatigability. Responses to verbal commands and questions required repetition. Dyscalculia was present.

A neurological examination revealed moderate amblyopia, with equal and reactive pupils bilaterally (D = S). Fine horizontal nystagmus was observed at the extremes of the lateral gaze, accompanied by reduced convergence. Decreased cutaneous sensation was noted in the distribution of the trigeminal nerve, consistent with facial hypaesthesia. Extraocular motor function was preserved, with a full, painless range of motion of the globes. No signs of bulbar or pseudobulbar dysfunction were identified. Muscle strength was preserved in all extremities, with symmetrical deep tendon reflexes (D = S). However, muscle tone was mildly reduced on the right side.

Significant gait and coordination disturbances were evident. The patient was unable to maintain balance during the Romberg test and demonstrated instability in multiple directions while ambulating, consistent with cerebellar ataxia. Finger-to-nose testing elicited intention tremor, further supporting cerebellar involvement. No meningeal signs or pathological reflexes were present. The patient’s affect and emotional stability remained intact throughout the assessment.

**Figure 1:**
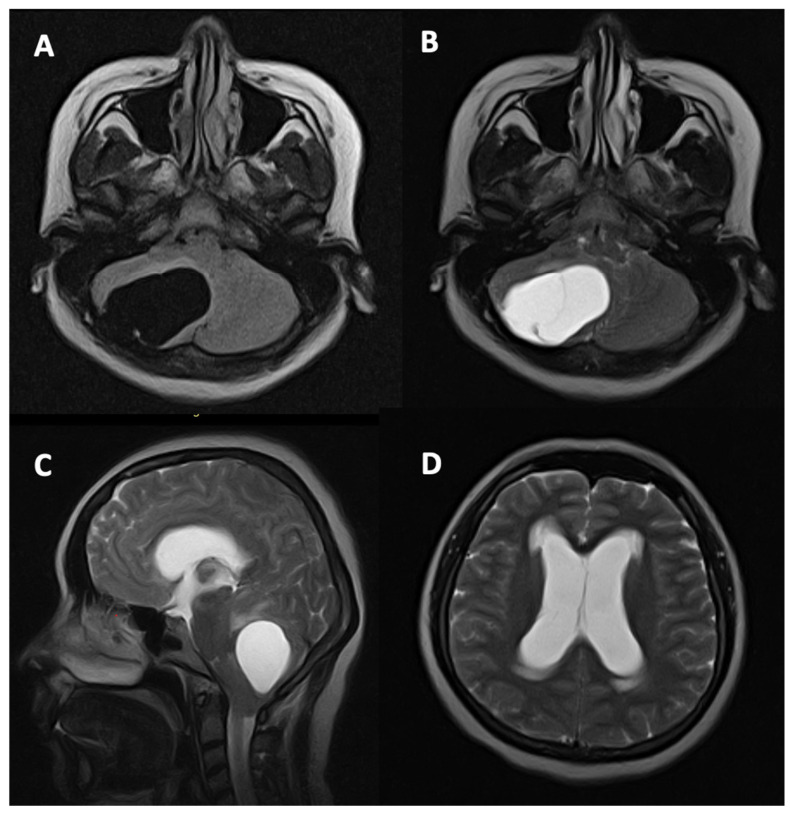
Preoperative MRI of the brain. Axial T1WI (**A**), axial T2WI (**B**), sagittal T2WI (**C**), and axial T2WI (**D**) demonstrated a well-circumscribed cystic lesion in the right cerebellar hemisphere measuring 5.61 × 4.24 × 3.45 cm, causing a significant mass effect with compression of the brainstem and fourth ventricle. Secondary obstructive triventricular hydrocephalus was present, with dilatation of the lateral and third ventricles and periventricular hyperintense signal changes on T2WI, consistent with transependymal cerebrospinal fluid flow (Evans Index—0.42). Inferior displacement of the cerebellar tonsils was observed, indicating elevated intracranial pressure.

MRI of the brain demonstrated a well-demarcated cystic lesion located in the right cerebellar hemisphere, causing compression of the fourth ventricle. This was associated with secondary obstructive triventricular hydrocephalus, evidenced by ventricular dilatation and periventricular hyperintensities on T2-weighted imaging. Inferior displacement of the cerebellar tonsils was also noted, indicative of elevated intracranial pressure (Figure 1).

In view of the patient’s progressive neurological deterioration and radiological findings suggestive of brainstem compression, an urgent multidisciplinary consultation was convened. The team comprised neurosurgeons, obstetricians, anaesthesiologists, and a neurologist. A comprehensive management strategy was formulated, prioritising timely neurosurgical intervention while ensuring maternal and fetal safety. Preparations were made for potential obstetric and perioperative complications.

### 2.3. Differential Diagnosis

The differential diagnosis for a cystic posterior fossa mass in a pregnant woman presenting with progressive cerebellar symptoms and obstructive hydrocephalus included pilocytic astrocytoma (PA), haemangioblastoma, ependymoma, and medulloblastoma. PA was the leading consideration, given its predilection for the cerebellar hemisphere, characteristic cystic morphology, and typically indolent growth pattern. Haemangioblastoma was initially suspected based on MRI findings; however, the absence of associated systemic features—such as polycythaemia or evidence of von Hippel–Lindau disease—made this diagnosis less likely. Ependymoma and medulloblastoma were also considered but were deemed less probable due to their typical imaging features, clinical progression, and lower likelihood in this age group and clinical context.

### 2.4. Management and Treatment

Given the progressive neurological decline and imaging evidence of brainstem compression and hydrocephalus, the patient was scheduled for urgent surgical intervention. A multidisciplinary team assessed and communicated the potential risks, including intraoperative death, haemorrhage, brainstem ischemia, cranial nerve injury, cerebrospinal fluid leak, infection, and bulbar syndrome.

The patient underwent a right-sided occipital craniotomy via a retrosigmoid approach for microsurgical resection of a cystic–solid lesion in the right cerebellar hemisphere. The procedure was guided by intraoperative neuronavigation. General endotracheal anaesthesia was administered, and continuous fetal monitoring was ensured using Holter cardiotocography. Following induction, the patient was positioned in the left lateral decubitus position with the head securely fixed in a Mayfield clamp. After multiple skin preparations with povidone-iodine solution, a linear skin incision approximately 8 cm in length was made, located 2 cm medial to the right mastoid notch. Suboccipital musculature was dissected to expose the occipital bone, and a craniectomy measuring approximately 3.4 × 4.8 cm was performed. The dura appeared tense and adherent to the inner surface of the bone, with no visible pulsations. To reduce intracranial pressure and facilitate safer brain retraction, 200 mL of 15% Mannitol and 8 mg of Dexamethasone were administered intravenously. Following osmotherapy, brain turgor visibly decreased, and the cerebellar surface appeared more relaxed and accessible upon opening the dura. The dura was incised in an X-shaped fashion under the operating microscope. Upon dural opening, a cystic–solid tumour was immediately encountered within the cerebellar hemisphere, located near the surface and displacing adjacent cerebellar tissue medially. The cyst component contained yellow, xanthochromic fluid, whereas the solid portion was soft, moderately vascular, and pink–yellow in appearance. The tumour displayed a clear plane of dissection and was well demarcated from the surrounding parenchyma, with no evidence of cortical infiltration. Adhesions to adjacent vasculature were minimal, and the tumour was not infiltrative, which facilitated en bloc resection. There was no significant adherence to major vessels or critical brainstem structures. Although the tumour was located in proximity to the brainstem, it remained well demarcated and superficially situated within the cerebellar hemisphere, with a clear dissection plane. Intraoperative neurophysiological monitoring was not available at the time of surgery due to temporary institutional resource limitations; however, the distinct anatomical boundaries and lack of brainstem infiltration allowed for safe microsurgical resection. Gross total resection was achieved using microsurgical techniques under continuous neuronavigation guidance. Both cystic wall and solid tumour components were submitted for histopathological examination. Haemostasis was secured using haemostatic agents, including Surgicel^®^ and absorbable gelatin sponges, with careful bipolar coagulation of bleeding surfaces. A watertight dural closure was performed, followed by reconstruction with autologous periosteum. The bone flap was intentionally not replaced to allow for postoperative decompression, given the risk of ongoing cerebellar oedema and raised intracranial pressure. Estimated intraoperative blood loss was approximately 200 mL. The surgery was completed without intraoperative complications, utilising a Karl Zeiss Pentero operating microscope and standard microneurosurgical instrumentation. The wound was irrigated, and an aseptic multilayer dressing was applied before extubation and transfer to the neurosurgical intensive care unit for postoperative monitoring.

### 2.5. Histopathological and Molecular Evaluation

A histological examination of the resected lesion, stained with haematoxylin and eosin, revealed fragments of the cerebellar cortex infiltrated by tumour tissue composed of bipolar and multipolar astrocytes with long cytoplasmic processes and abundant glial fibrils. Numerous Rosenthal fibres—eosinophilic corkscrew-shaped structures—were identified, along with eosinophilic granular bodies. Focal areas showed a mesh-like arrangement of small astrocytes with scant cytoplasm, and there were regions of glomeruloid vascular proliferation and large cystic spaces. The pathomorphological features were consistent with PA, CNS WHO Grade I. Further molecular studies were recommended to assess for KIAA1549::BRAF gene fusion, BRAF V600E mutation, and H3 K27M mutation, but the results of the tests were not available at the time of diagnosis. Molecular profiling was not performed in this case due to resource limitations. Nevertheless, the diagnosis of pilocytic astrocytoma was established based on characteristic histopathological findings, which remain the cornerstone of diagnosis in the absence of molecular data.

### 2.6. Postoperative Imaging and Fetal Assessment

**Figure 2:**
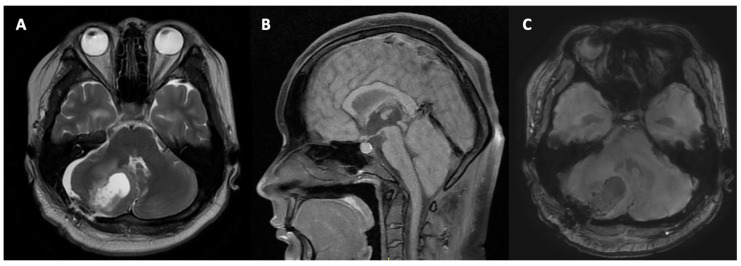
Postoperative MRI (day 1). Axial T2WI (**A**), sagittal T1WI (**B**), and axial 3D eSWAN (**C**) obtained 1 day after surgery demonstrated adequate decompression of the posterior fossa with restoration of fourth ventricle patency. No residual cystic lesion was identified. Early regression of ventricular dilatation was noted, without radiological signs of acute postoperative complications.

Magnetic resonance imaging performed on the second day post-op revealed a postoperative resection cavity in the right cerebellar hemisphere (Figure 2). The cavity exhibited signal characteristics consistent with cerebrospinal fluid. Surrounding the surgical site, there was evidence of perifocal brain oedema. Diffusion-weighted imaging and apparent diffusion coefficient sequences demonstrated areas of restricted diffusion along the resection margin, suggestive of focal postoperative ischaemic changes. Additionally, mucoependymal oedema was identified in the periventricular white matter bilaterally. Inferior displacement of the cerebellar tonsils by approximately 5–6 mm below the Chamberlain line was noted, consistent with mild tonsillar herniation. Ventricular imaging showed dilation of the lateral and third ventricles (Evans Index: 29%), with compression of the fourth ventricle. However, the patient remained clinically stable, and these findings did not necessitate cerebrospinal fluid diversion via external ventricular drainage or shunting at that time. The decision to proceed directly with tumour resection without preoperative ventricular drainage was based on the rapid neurological decline and the multidisciplinary consensus that tumour decompression would likely relieve the obstructive component of the hydrocephalus. Mild soft tissue oedema was observed in the subcutaneous fat at the craniotomy site, with no features suggestive of infection or wound complication.

In the immediate postoperative period, the patient was admitted to the neurosurgical intensive care unit for the first 24 h. During this time, fetal well-being was monitored twice daily using cardiotocography, in addition to continuous maternal haemodynamic and neurological surveillance. No abnormalities in fetal heart rate patterns or uterine activity were detected. From postoperative day 2 onward, as the maternal condition remained clinically stable, CTG monitoring was performed once daily in accordance with institutional obstetric protocols. No signs of fetal distress were observed. Given the stable maternal and fetal status, further intensive fetal monitoring was not deemed necessary. A fetal ultrasound performed on postoperative day 5, corresponding to a gestational age of 25 weeks and 1 day, demonstrated a viable fetus in breech presentation with no structural abnormalities or signs of distress.

Following surgery, the patient was monitored closely in a multidisciplinary setting, including neurosurgical, obstetric, and intensive care teams. Her neurological condition showed progressive improvement over the early postoperative period, with the stabilisation of consciousness, the resolution of vomiting, and gradual improvement in coordination and ambulation. Intracranial pressure remained stable without the need for further surgical intervention.

### 2.7. Postoperative Follow-Up and Delivery Outcomes

The patient was discharged in a stable condition on the 5th day after the operation and subsequently followed on an outpatient basis by a multidisciplinary team including a neurosurgeon, neurologist, and obstetrician–gynecologist. At 38 weeks and 2 days of gestation, the patient was admitted to a tertiary maternal and child health care center due to the premature rupture of membranes and subsequently underwent an urgent caesarian section. The postoperative and postpartum courses were uneventful, and the maternal condition remained stable. A live full-term male neonate was delivered with a birth weight of 3100 g, length of 54 cm, head circumference of 36 cm, and chest circumference of 35 cm. Apgar scores were 8 and 9 at 1 and 5 min, respectively. The early neonatal period was uncomplicated, with no congenital anomalies or neurological abnormalities identified. The newborn remained in rooming-in care with the mother and was discharged in satisfactory condition.

**Figure 3:**
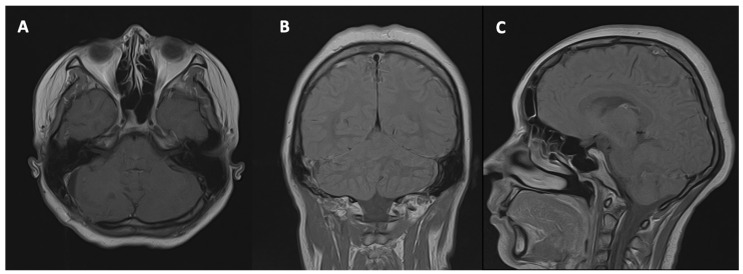
Follow-up MRI at 6 months after surgery. Axial T1-weighted imaging with contrast (**A**), coronal T1-weighted imaging with contrast (**B**), and sagittal T1-weighted imaging with contrast (**C**) demonstrated stable postoperative changes without evidence of residual or recurrent tumour. The posterior fossa structures were preserved, with normal configuration of the fourth ventricle and no signs of hydrocephalus or mass effect.

Six-month postoperative magnetic resonance imaging demonstrated post-surgical changes consistent with prior tumour resection in the right cerebellar hemisphere. A residual cystic cavity was present at the surgical site, without evidence of residual tumour tissue, local recurrence, or intracranial metastasis. No hydrocephalus or significant mass effect was observed (Figure 3). These findings were interpreted as consistent with a stable postoperative course and no signs of disease progression.

**Figure 4:**
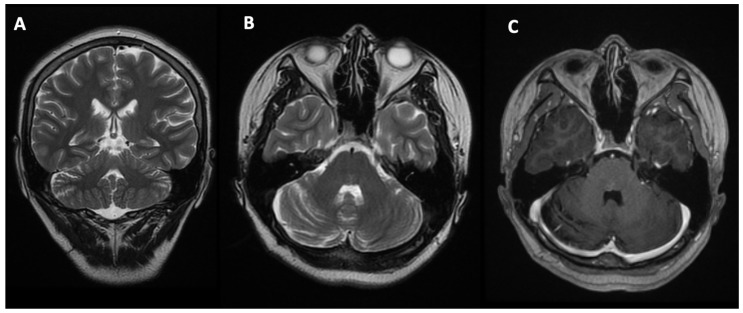
Follow-up MRI at 12 months after surgery. Coronal T2-weighted imaging (**A**), axial T2-weighted imaging (**B**), and post-contrast 3D T1-weighted imaging (3D AX BRAVO) (**C**) demonstrated stable postoperative findings with no evidence of tumour recurrence. The posterior fossa structures and cerebrospinal fluid pathways were preserved, with no signs of hydrocephalus or mass effect.

As of the current follow-up period—approximately 12 months postoperatively—the patient remains neurologically stable, without the recurrence of symptoms. Routine imaging (Figure 4) and clinical surveillance continue, with no new concerns identified. The child demonstrates normal growth and neurodevelopment appropriate for their age, with no reported neurological, developmental, or somatic abnormalities during routine paediatric follow-up.

## 3. Discussion

PA is predominantly recognised as a paediatric brain tumour, representing a substantial proportion of CNS neoplasms in children and constituting the most commonly diagnosed glioma in this population [8]. Conversely, its occurrence in adults is rare, accounting for merely 2% of all adult intracranial tumours [9]. This rarity is further accentuated in the context of pregnancy. A large retrospective cohort study using data from the Nationwide Inpatient Sample (NIS) identified 437 cases of benign intracranial neoplasms among approximately 19 million pregnancy-related hospitalisations; however, no specific data pertaining to PAs were reported within this cohort [3]. Thus, precise epidemiological figures regarding the prevalence of PA in pregnant women remain unavailable. In general, CNS tumours during pregnancy are uncommon, and published data concerning their incidence, management strategies, and clinical outcomes remain sparse.

In adults, PAs most frequently originate in the posterior cranial fossa, particularly within the cerebellar hemispheres and vermis, situating them in close proximity to vital neuroanatomical structures such as the fourth ventricle and brainstem [8]. The confined nature of the posterior fossa, combined with the critical functional importance of its contents, renders these tumours especially hazardous. PAs are most commonly found in the cerebellum—comprising approximately 42–60% of cases—and also occur with notable frequency in the optic pathways, hypothalamus, and brainstem [10,11]. Within this restricted anatomical compartment, even relatively small lesions may exert a substantial mass effect, potentially resulting in obstructive hydrocephalus, brainstem compression, and rapid neurological decline. Recent MRI-based analyses indicate that posterior fossa pilocytic astrocytomas typically appear hypointense on T1-weighted images and hyperintense on T2/FLAIR sequences, with variable contrast enhancement ranging from minimal to nodular patterns. In brainstem and cerebellar locations, lesions may demonstrate either focal or partially infiltrative features, sometimes mimicking higher-grade gliomas [12,13]. Advanced MRI techniques may further assist in differentiation. Diffusion-weighted imaging can show restricted diffusion in selected cases, while perfusion imaging generally demonstrates lower relative cerebral blood volume compared with high-grade gliomas, reflecting the typically low proliferative index of PA [12]. In this context, conventional MRI findings should always be interpreted in conjunction with clinical presentation and anatomical location. In pregnant patients in particular, where contrast administration may be limited, a careful assessment of the lesion morphology, mass effect, and surrounding edema becomes even more critical for surgical planning. In our case, the well-demarcated cystic–solid configuration and significant brainstem compression were sufficient to justify urgent intervention despite the absence of contrast-enhanced sequences, underscoring that radiological decision-making must ultimately be guided by clinical urgency and multidisciplinary evaluation rather than imaging characteristics alone. MRI without contrast is the preferred imaging modality for evaluating intracranial tumours during pregnancy, as it avoids ionising radiation and is considered safe at any gestational stage while providing high diagnostic accuracy [2,14,15]. Existing guidelines indicate no evidence of fetal harm, even with first-trimester exposure [16], and in clinical practice, non-contrast MRI is used in the majority of pregnant patients with brain tumours (83%) [17]. Gadolinium-based contrast agents are reserved for strict indications. Large population data have suggested an association between gadolinium exposure and adverse fetal outcomes [15,18], leading current recommendations to limit its use to situations in which non-contrast imaging is insufficient [18]. Cranial CT is considered a secondary modality, primarily for emergency conditions such as suspected haemorrhage; although fetal radiation exposure is low, its use—particularly in early pregnancy—should remain restricted [16,17].

Astrocytomas, encompassing both pilocytic and diffuse subtypes, have been observed to exhibit accelerated growth during pregnancy. This phenomenon is most commonly attributed to haemodynamic alterations characteristic of the gravid state—specifically, hypoxaemia, hypercapnia, and hypervolaemia—which may exacerbate cerebral oedema and unmask the clinical manifestations of the tumour [4]. In parallel, hormonal influences have also been implicated, with particular attention given to prolactin. Through its interaction with the prolactin receptor (PRLR), prolactin may activate intracellular signalling cascades, including the JAK2/STAT5 and MAPK/ERK pathways, potentially promoting tumour cell proliferation [7]. Therefore, the progression of astrocytomas during pregnancy is likely driven by a complex interplay of hormonal (e.g., progesterone, prolactin), vascular, and systemic haemodynamic factors. Nonetheless, definitive mechanistic data specific to astrocytomas remain lacking. Current understanding is largely derived from indirect clinical observations and extrapolation from studies investigating other glioma subtypes.

According to several case analyses, surgical intervention remains the cornerstone of treatment for posterior cranial fossa tumours during pregnancy. In instances of significant brainstem compression and obstructive hydrocephalus, tumour resection is considered an urgent, life-saving procedure to prevent catastrophic neurological complications [19].

With respect to PA, only a limited number of cases diagnosed during pregnancy have been documented in the literature. Umehara et al. [20] reported a case of brainstem PA in a pregnant patient whose tumour remained stable during her first pregnancy but demonstrated rapid growth at 25 weeks’ gestation during her second. An emergency partial resection was performed, and the pregnancy was preserved, resulting in the live birth of the child. Remarkably, the tumour volume decreased spontaneously by nearly 50% postpartum, suggesting a possible hormonal influence on tumour behaviour during gestation. Similarly, Schmidt and Hanna [21] described a patient with neurofibromatosis type 1 and a long-standing cerebellar PA, which had remained stable for over a decade. At 24 weeks’ gestation, she experienced sudden tumour progression accompanied by intratumoural haemorrhage, obstructive hydrocephalus, and tonsillar herniation. Despite emergency posterior fossa decompression, haematoma evacuation, and partial tumour resection, the neurological deterioration was irreversible, and the outcome was fatal. Another case, reported by Trager et al. [22], involved a 31-year-old woman at 38 weeks of gestation presenting with progressive headaches and seizures. MRI revealed a posterior fossa tumour, prompting an emergency Caesarean section followed by craniotomy and tumour excision. Histopathological analysis confirmed a PA. The combined obstetric and neurosurgical approach facilitated a favourable outcome for both mother and infant. Collectively, these isolated case reports illustrate the highly variable behaviour of PA during pregnancy, ranging from relative stability to rapid progression or malignant transformation necessitating urgent intervention. According to findings by Yust-Katz et al. [23], PAs and other World Health Organization grade I gliomas generally remain stable throughout pregnancy. In contrast, 44% of patients with grade II–III gliomas experienced tumour progression either during gestation or within eight weeks postpartum, including cases of malignant transformation. These observations underscore the critical importance of rigorous MRI surveillance and the pursuit of maximal safe resection in managing posterior cranial fossa tumours during pregnancy. Pilocytic astrocytoma during pregnancy poses a unique dilemma, particularly when located in the posterior fossa. Although PA is a WHO grade I tumour with generally favourable biology, its anatomical confinement within the posterior cranial fossa may lead to obstructive hydrocephalus and brainstem compression, necessitating urgent intervention regardless of histological grade. In contrast to supratentorial tumours in pregnant women reported by Filippi et al. [24] and Oblitas López et al. [25], which primarily presented with seizures and allowed more controlled surgical timing, posterior fossa lesions were frequently associated with a mass effect and neurological deterioration [26]. Despite the potentially aggressive nature of certain intracranial tumours, favourable outcomes are achievable when a diagnosis is made early and managed through a multidisciplinary approach. In cases where no evidence of tumour progression is observed following surgery, pregnancy may be safely prolonged to term. One such example involves a patient with medulloblastoma who underwent an elective Caesarean section at 37 weeks’ gestation [19]. Furthermore, vaginal delivery is not contraindicated per se, provided the neurological status remains stable and there is no associated risk of elevated intracranial pressure. The coexistence of low-grade histology and high-risk anatomical location therefore represents a paradox: biologically favourable yet surgically urgent. Our case supports the concept that timely gross total resection during the second trimester, within a multidisciplinary framework, can result in favourable maternal and fetal outcomes despite this inherent complexity. A structured comparative overview of previously reported cases, including clinical characteristics, management strategies, and outcomes, is presented in Table 1.

The present case illustrates a rare yet instructive example of the successful neurosurgical management of a posterior fossa PA during the second trimester of pregnancy, without necessitating preterm delivery. At 23 weeks and 5 days of gestation, the patient presented with signs of progressive brainstem compression and obstructive hydrocephalus, warranting an urgent retrosigmoid craniotomy and gross total resection under general anaesthesia, accompanied by continuous intraoperative fetal monitoring. Although low-grade gliomas such as PA are generally associated with favourable long-term prognoses, pregnancy introduces unique physiological stresses and a heightened risk of acute neurological deterioration. These factors necessitate vigilant clinical surveillance and prompt, coordinated intervention. This case emphasises that in scenarios involving maternal neurological decline and incomplete fetal maturity, timely neurosurgical intervention may be safely and effectively executed within a multidisciplinary framework, thereby optimising outcomes for both mother and child.

## 4. Conclusions

PA is an infrequent diagnosis in adult patients and an exceptional finding during pregnancy. When localised to the posterior cranial fossa, these tumours may cause rapid neurological deterioration due to brainstem compression and obstructive hydrocephalus, necessitating urgent neurosurgical intervention. This case highlights the diagnostic and therapeutic complexity posed by such tumours in pregnant patients, in whom symptoms overlap with common gestational complaints, which may delay recognition.

Our experience demonstrates that the gross total resection of a posterior fossa PA can be safely achieved during the second trimester of pregnancy when conducted within a multidisciplinary framework incorporating neurosurgery, obstetrics, anaesthesiology, and neonatology. Close perioperative monitoring, appropriate anaesthetic management, and intraoperative fetal surveillance are critical to safeguarding both maternal and fetal outcomes.

Although PAs are considered low-grade gliomas, their clinical course during pregnancy can be unpredictable, possibly influenced by haemodynamic and hormonal changes. Timely neuroimaging and decisive surgical management remain essential in cases of neurological decline. This case reinforces the principle that pregnancy, in itself, should not preclude potentially life-saving neurosurgical treatment, and with appropriate planning, favourable maternal and neonatal outcomes can be achieved.

## Figures and Tables

**Table 1:** Reported cases of pilocytic astrocytoma and other posterior fossa tumours managed during pregnancy.

	Current Study	[24]	[25]	[27]	[26]	[28]
**Maternal age**	24	34	33	35	Late adolescence	23
**G/P**	G1P0	G3P2	N/A	Primipara	Multiparous	N/S
**G/A**	23 + 5 w	27 + 5 w	18 w	18 w	~5 w	8 w
**Trimester**	II	III	II	II	I	I
**Obstetric complications**	None	None	N/A	N/A	N/A	TOP
**Tumour location**	Cerebellum	Parietal	Frontal	Temp-parietal	CPA	Cerebellum
**Tumour size**	Large cystic	5 cm	47–53 mm	Large	3.8–4.9 cm	Large
**Hydrocephalus**	Yes	No	No	Yes	Yes	N/S
**Brainstem compression**	Yes	No	No	No	Yes	Mass effect
**Histology**	PA (WHO I)	IDH astro II–III	Astro II	GBM IV	Meningioma I	Capillary haemangioma
**Molecular data**	N/P	IDH+, MGMT+	N/R	IDH1+, ATRX-	N/R	N/R
**Main present symptoms**	HA, ataxia	Seizure	Seizure	Aphasia	HA, vertigo	HA, visual
**Neurological deterioration**	Yes	Yes	Yes	Yes	Yes	Yes
**Timing of surgery**	During pregnancy	During pregnancy	During pregnancy	During pregnancy	During pregnancy	After TOP
**Type of surgery**	GTR	STR + Second-stage awake resection postpartum	STR	Resection	Tumour debulking	STR (Stage I) → GTR (Stage II)
**Use of IONM**	No	Yes	Yes	N/S	Yes	N/A
**Use of fetal monitoring**	Yes	Yes	Yes	Yes	Yes	N/A
**Mode of delivery**	CS 38 w	CS 37 w	CS 37 w	CS 26 w	CS 38 w	TOP
**Maternal outcome**	Improved	Stable	Stable	Deceased 4 m after surgery	Improved	Improved
**Fetal outcome**	Live term	Live	Live	Live	Live	TOP
**Follow-up duration**	12 m	4 m	9 m	4 m	N/A	12 m
**Recurrence**	No	Residual	Residual	Yes	N/R	Yes

G—gravida; P—para; w—weeks; T—trimester; N/A—not applicable(parameter not relevant to the specific case); N/S—not specified(information mentioned but not detailed); N/R—not reported (no data provided in the source); N/P—not performed (procedure or assessment was not carried out); TOP—termination of pregnancy; PA—pilocytic astrocytoma; Astro—astrocytoma; GBM—glioblastoma; CPA—cerebellopontine angle; HA—Headache; GTR—gross total resection; STR—subtotal resection; IONM—intraoperative neurophysiological monitoring; CS—Caesarean section; m—months.

## Data Availability

All data supporting the findings of this study are available upon request from the National Centre for Neurosurgery in Astana, Kazakhstan. Data will be provided in compliance with confidentiality and ethical standards.

## Abbreviations

The following abbreviations are used in this manuscript:

D	Dexter (right side)
S	Sinister (left side)
T1WI	T1-weighted imaging
T2WI	T2-weighted imaging
3D eSWAN	Three-dimensional enhanced susceptibility-weighted angiography
PA	Pilocytic astrocytoma
LGG	Low-grade glioma
PRLR	Prolactin receptor
JAK2/STAT5	Janus kinase 2/Signal transducer and activator of transcription 5
MAPK/ERK	Mitogen-activated protein kinase/Extracellular signal-regulated kinase
KIAA1549::BRAF	KIAA1549–BRAF gene fusion (common genetic alteration in pilocytic astrocytoma)
BRAF V600E	BRAF gene mutation resulting in valine-to-glutamic acid substitution at codon 600
H3 K27M	Histone H3 lysine 27-to-methionine mutation
CTG	Cardiotocography (fetal heart rate monitoring)
